# Anti-inflammatory activity of aqueous extract and bioactive compounds identified from the fruits of *Hancornia speciosa* Gomes (Apocynaceae)

**DOI:** 10.1186/s12906-016-1259-x

**Published:** 2016-08-05

**Authors:** Manoela Torres-Rêgo, Allanny Alves Furtado, Mariana Angélica Oliveira Bitencourt, Maira Conceição Jerônimo de Souza Lima, Rafael Caetano Lisbôa Castro de Andrade, Eduardo Pereira de Azevedo, Thaciane da Cunha Soares, José Carlos Tomaz, Norberto Peporine Lopes, Arnóbio Antônio da Silva-Júnior, Silvana Maria Zucolotto, Matheus de Freitas Fernandes-Pedrosa

**Affiliations:** 1 Departamento de Farmácia, Laboratório de Tecnologia e Biotecnologia Farmacêutica (TecBioFar), Faculdade de Farmácia, Centro de Ciências da Saúde, Universidade Federal do Rio Grande do Norte, Rua Gal. Gustavo Cordeiro de Farias, s/n, Petrópolis, CEP 59012-570 Natal, RN Brazil; 2 Departamento de Pós-graduação em Biotecnologia, Universidade Potiguar, Avenida Senador Salgado Filho, 1610, Lagoa Nova, CEP 59056-000 Natal, RN Brazil; 3 Departamento de Farmácia, Laboratório de Farmacognosia, Faculdade de Farmácia, Centro de Ciências da Saúde, Universidade Federal do Rio Grande do Norte, Rua Gal. Gustavo Cordeiro de Farias, s/n, Petrópolis, CEP 59012-570 Natal, RN Brazil; 4 Departamento de Física e Química, Núcleo de Pesquisas em Produtos Naturais e Sintéticos (NPPNS), Faculdade de Ciências Farmacêuticas de Ribeirão Preto, Universidade de São Paulo, Avenida do Café, s/n, Monte Alegre, CEP 14040-903 Ribeirão Preto, SP Brazil

**Keywords:** Anti-inflammatory, Apocynaceae, Chlorogenic acid, *Hancornia speciosa*, *Mangabeira*, Rutin

## Abstract

**Background:**

*Hancornia speciosa* Gomes (Apocynaceae), popularly known as “mangabeira,” has been used in folk medicine to treat inflammatory disorders, hypertension, dermatitis, diabetes, liver diseases and gastric disorders. Although the ethnobotany indicates that its fruits can be used for the treatment of ulcers and inflammatory disorders, only few studies have been conducted to prove such biological activities. This study investigated the anti-inflammatory properties of the aqueous extract of the fruits of *H. speciosa* Gomes as well as its bioactive compounds using in vivo experimental models.

**Methods:**

The bioactive compounds were identified by High Performance Liquid Chromatography coupled with diode array detector (HPLC-DAD) and Liquid Chromatography coupled with Mass Spectrometry (LC-MS)*.* The anti-inflammatory properties were investigated through in vivo tests, which comprised xylene-induced ear edema, carrageenan-induced peritonitis and zymosan-induced air pouch. The levels of IL-1β, IL-6, IL-12 and TNF-α were determined using ELISA.

**Results:**

Rutin and chlorogenic acid were identified in the extract as the main secondary metabolites. In addition, the extract as well as rutin and chlorogenic acid significantly inhibited the xilol-induced ear edema and also reduced the cell migration in both carrageenan-induced peritonitis and zymosan-induced air pouch models. Reduced levels of cytokines were also observed.

**Conclusion:**

This is the first study that demonstrated the anti-inflammatory activity of the extract of *H. speciosa* fruits against different inflammatory agents in animal models, suggesting that its bioactive molecules, especially rutin and chlorogenic acid are, at least in part, responsible for such activity. These findings support the widespread use of *Hancornia speciosa* in popular medicine and demonstrate that its aqueous extract has therapeutical potential for the development of herbal drugs with anti-inflammatory properties.

## Background

The use of plants is a common practice in Brazil’s folk medicine and it has been based on the build-up of empirical knowledge by various ethnic groups about the therapeutic effect of these herbal plants [1]. These medicinal plants are widely used for research of new drugs as they represent a rich source of compounds with pharmacological properties [2]. Since these plants are naturally found in relative abundance in Brazil, the population has easy access to these natural sources at relatively low cost and minimal side effects [3].

*Hancornia speciosa* Gomes, which belongs to the family of Apocynaceae and is popularly known as “mangabeira”, is a fruit plant species native to Brazil [4]. It is found in the cerrado, caatinga and savanna vegetation [5]. In traditional medicine, its fruits have been used to treat ulcers, tuberculosis and inflammatory disorders [6], whereas the infusion of barks have been used for treating gastric ulcers, stomach disturbances and inflammatory processes [7]. In addition, its roots and leaves are used to treat high blood pressure and rheumatism [8, 9]. *Hancornia speciosa* leaves have been demonstrated to exhibit anti-hypertensive [10–12], anti-carcinogenic [13, 14] and anti-diabetic properties [15]. Its barks have a marked gastroprotective effect and anti-*Helicobacter pylori* activity [16, 17]. However, there have been few studies that reported the pharmacological effects of *Hancornia speciosa* fruits and just recently, a group described the antimutagenic potential of its fruit pulp [18]. Its latex has shown anti-inflammatory effect through reduction of edema induced by bradykinin, histamine and serotonin, as well as by inhibiting inflammation induced by subcutaneous carrageenan injection and through inhibition of leukocytes migrations, nitric oxide, PGE2 and cytokines production in mice [19]. Therefore, the anti-inflammatory effect of *Hancornia speciosa* latex corroborated the popular use of its fruits for the treatment of ulcers and inflammatory disorders.

Inflammation is an immune system response triggered by different stimuli, including chemical, physical and biological [20]. The recognition of a harmful agent or stimulus triggers the activation and amplification of the immune response, resulting in cell activation and release of various mediators responsible for the inflammatory response [21]. Among these mediators, cytokines (IL-1β, IL-6, IL-12 and TNF-α) are noteworthy as they are responsible for inducing the expression of adhesion molecules and for inducing leukocytes sequestration from the blood stream towards the inflammation site [22]. Therefore, when the inflammatory process is not controlled, it can cause tissue damage [23]. In order to control the inflammation process, drugs that inhibit this clinical condition, such as non-steroidal anti-inflammatory and steroidal anti-inflammatory drugs are used. However, the overuse of non-steroidal anti-inflammatory drugs can cause side-effects such as gastrointestinal and cardiovascular complications [24], whereas the steroidal anti-inflammatory drugs may cause reduced resistance to infections, aggravation of ulcers and osteoporosis [25]. On the other hand, studies have shown that plants are able to reduce the inflammatory process with considerably less side-effects. For instance, some vegetal extracts are able to reduce the total number of leukocytes, to decrease the secretion of cytokines and histamine, as well as to reduce the proliferation of lymphocytes [26, 27]. Given the importance of these biological properties, traditional medicinal knowledge along with modern techniques have optimized the process of drug discovery from medicinal plants [28, 29].

Based on the fact that only few studies have reported the pharmacological properties of *H. speciosa* fruits, despite its widespread popular use as an anti-inflammatory agent, this work aims to investigate the chemical constituents of these fruits and associate its major compounds with the anti-inflammatory effect. Therefore, phytochemical analysis of the aqueous extract of the fruits of *H. speciosa* was performed in order to identify the bioactive compounds, followed by the evaluation of its anti-inflammatory activity in experimental models using mice. The in vivo models were the (i) carrageenan-induced peritonitis, (ii) xylene-induced ear edema, and (iii) zymosan-induced air pouch model, where the (iv) production of cytokines (IL-1β, IL-6, IL-12 and TNF-α) was also evaluated. To the best of our knowledge, this is the first study that investigated the anti-inflammatory effect of the extract of the fruits of *H. speciosa*.

## Methods

### Plant material

The mature fruits were collected at Barra do Punaú region (lat:-5,34527 long:-35,41666), Rio do Fogo City, Rio Grande do Norte, Brazil, in March of 2011 and was identified by botanist Dr. Jomar Gomes Jardim. A voucher specimen (UFRN 16880) was deposited in the Herbarium of the Federal University of Rio Grande do Norte, Brazil. The collection of the plant material was conducted under authorization of the Brazilian Authorization and Biodiversity Information System (SISBIO) (process number 35017) and Brazilian Access Authorization and Dispatch Component of Genetic Patrimony (Process 010844/2013-9).

### Preparation of the aqueous extract

Fresh fruits (500 g) were sliced in pieces and submitted to extraction by decoction using hot water at 100 °C (1:10, w:v, plant:solvent) for 15 min. Further, the extract was filtered through Whatman paper no.1 and lyophilized. The final dried mass was 2.5 g (yield around 0.5 %).

### High Performance Liquid Chromatography coupled with diode array (HPLC-DAD) Analysis

*A*nalyses were performed using HPLC Varian® Pro Star 335 with diode array detector. A Phenomenex® RP-18 column (250 x 4.6 mm, 5 μm) was used. The eluents were: (A) acetonitrile and (B) water with 0.3 % acetic acid, with the following gradient (v/v): 13 %, 0–5 min (B); 13–18 %, 5–25 min (B); 18–20 %, 25–30 min (B); 20–21 %, 30–35 min (B). Flow elution was 0.7 mL/min and 20 μL of each sample was injected. The lyophilized extract and standard solutions were resuspensed in methanol: water, 1:1 (v/v) and the final concentrations were 5 mg/mL and 50 μg/mL. The chromatogram was visualized in 270 and 340 nm, where each peak and their retention time (*R*_*t*_) was compared with those of the standards. The peaks that showed similar UV and *R*_*t*_ were analyzed by co-injection (reference standard + extract) with the purpose of observing any increase in the peak area. Samples and solvents were previously filtered through a 0.45 μm membrane and degassed. Both rutin (purity 94 %) and chlorogenic acid (purity 95 %) were purchased from Sigma-Aldrich®, USA.

### Liquid Chromatography coupled with mass spectrometry (LC-MS) analysis

High resolution analyses by LC-MS were performed on a Shimadzu LC-20 AD apparatus equipped with an autosampler (SIL-20A, Shimadzu), diode array detector (SPD-M20AV, Shimadzu) and coupled with a micrOTOFII (Bruker Daltonics) ESI-qTOF mass spectrometer. The LC conditions were the same applied at High Performance Liquid Chromatography coupled with diode array (HPLC-DAD) Analysis. The column eluent was split at a ratio of 7:3, where the larger flow went to the DAD detector and the lower one went to the mass spectrometer.

Low resolution applied a similar Shimadzu LC-20 AD apparatus coupled with an ESI-ion trap mass spectrometer (amaZon, Bruker Daltonics). Again, the LC conditions were the same as described on High Performance Liquid Chromatography coupled with diode array (HPLC-DAD) Analysis. The column eluent was split at a ratio of 7:3, where the larger flow went to the DAD detector and the lower one went to the mass spectrometer.

### Animals

Male and female *Swiss* and BALB/c mice (25–35 g), 6–8 weeks of age, were maintained at a temperature of 22 ± 2 °C and at a 12/12 h light/dark cycle. Each test group was composed of five animals (*n* = 5). The experimental protocol was approved by the Committee for Ethics in Animal Experimentation of the Universidade Federal do Rio Grande do Norte, Brazil, at the Protocol N° 008/2011 and in accordance with the guidelines of National Council for the Control of Animal Experimentation (CONCEA).

### Carrageenan-induced peritonitis model

Peritoneal inflammation was induced according to the procedure previously described in [30] with few modifications. BALB/c mice were inoculated intravenously (i.v.) with 100 μL of saline, aqueous extract of *Hancornia speciosa* fruits (20, 30 or 40 mg/kg), chlorogenic acid (2, 2.5 or 5 mg/kg) or rutin (2, 2.5 or 5 mg/kg), while dexamethasone was injected intraperitoneally (i.p.) (0.5 mg/kg) and used as the anti-inflammatory reference drug. After 30 min, the animals received carrageenan (1 mg/mL) or saline intraperitoneally. After 4 h, the animals were euthanized with an overdose of xylazine and ketamine (10 mg/kg–100 mg/kg) and peritoneal exudates were harvested by peritoneal lavage using 2 mL of saline, followed by centrifugation at 250 *g* for 10 min at 4 °C. Leucocytes count was determined using a Neubauer chamber [31, 32]. The supernatants were collected for determination of IL-1β, IL-6, IL-12 and TNF-α levels using an ELISA kit (eBioscience, USA) following the manufacturer’s instructions.

### Xylene-induced ear edema model

BALB/c mice were treated intraperitoneally with 100 μL of saline, dexamethasone (0.5 mg/kg), aqueous extract of *Hancornia speciosa* fruits (40, 50 or 60 mg/kg), rutin (2.5, 5 or 10 mg/kg) and chlorogenic acid (10, 12.5 or 15 mg/kg). Thirty minutes after the treatment, all the animals received 40 μL of xylene administered in the anterior and posterior surfaces of the right ear. The left ear was taken as control where only saline was administered. Fifteen minutes after xylene administration, the animals were euthanized and both ears were cut off at circular sections of 7 mm using a cork borer and then weighed [33]. The edematous response was measured as the weight difference between the right and left ears, where the inhibition level was then calculated as:

$$ \mathrm{Inhibition}\ \left(\%\right)=\left[1-\mathrm{E}\mathrm{t}/\mathrm{E}\mathrm{c}\right]\times 100, $$

where *Et* and *Ec* are the average weight of the edemas in the sample-treated and control groups, respectively.

### Zymosan-induced air pouch model

*Swiss* mice received 5 mL of sterile air subcutaneously (s.c.), which were injected into the back of the animals. After three days, 2.5 mL of sterile air was injected into the cavity. Six days after the initial air injection, the animals received intraperitoneal injection of saline, dexamethasone (2.0 mg/kg), aqueous extract of *Hancornia speciosa* fruits (40, 50, or 60 mg/kg), rutin (2.5, 5 or 10 mg/kg) and chlorogenic acid (10,12.5 or 15 mg/kg) [33]. After 30 min, zymosan solution (1 mg/mL) was injected into the air pouch. At pre-determined time points (6, 24, and 48 h), the animals were euthanized and exudates were harvested from each air pouch by washing with 2 mL of saline. Leucocytes count was determined using a Neubauer chamber [34–36]. The cell *pellet* was diluted in 500 mL of saline and the cell subpopulations count (polymorphonuclear and mononuclear cells) was determined based on the count of 100 cells using a hemocytometer [37].

### Statistical analysis

Data are expressed as mean ± standard deviation. Statistical analyses were performed by One-way ANOVA with Tukey’s test and regression analyses were performed using GraphPad Prism version 5.00 (San Diego, CA, USA). A difference in the mean values of *P* < 0.05 was considered as statistically significant.

## Results

### HPLC-DAD analysis

**Fig. 1:**
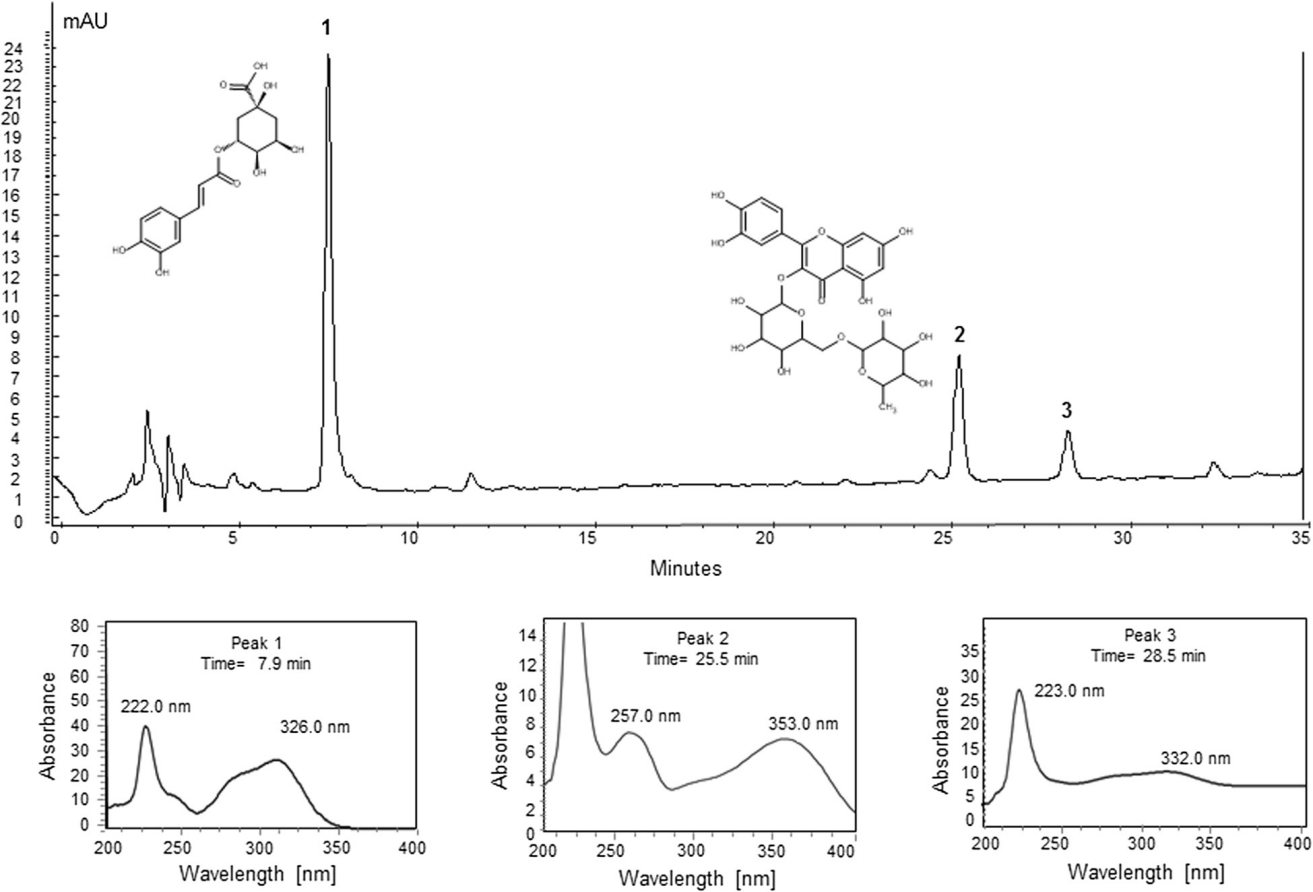
HPLC-DAD chromatograms of the aqueous extract of the fruits of *Hancornia speciosa*. The analysis shows three major peaks, designated as peak 1 (Rt = 7.9 min, solvent A: 87-82 %), peak 2 (Rt = 25.5 min, solvent A: 82-80 %) and peak 3 (Rt = 28.5 min, solvent A: 82-80 %), which correspond to chlorogenic acid, rutin and an unidentified phenolic acid compound, respectively. Stationary phase: column Phenomenex-Luna® C-18 (4.6 mm x 250 mm, 5 μm); mobile phase: gradient of acetonitrile: water with 0.3 % acetic acid; flow rate was kept constant at 1.0 ml/min; Detection UV of 340 nm

Analysis by HPLC-DAD showed the presence of chlorogenic acid (peak 1) and rutin (peak 2) in the aqueous extract of *Hancornia speciosa* fruit (Fig. 1). The standard solutions of these compounds were analyzed, showing retention times of 7.9 (solvent A: 87-82 %) and 25.5 min (solvent A: 82-80 %), respectively, which are similar to peaks 1 and 2. In addition, UV spectrum of peaks 1 and 2 exhibited UV *λ*_*max*_ of 222 and 326 nm (peak 1) and 257 and 353 nm (peak 2), respectively, which were similar to the UV spectrum of chlorogenic acid and rutin. Through the co-injection analysis of the standards and the aqueous extract, an increase in the peak areas was observed, confirming the presence of these compounds*.* Although peak 3 was not identified, the UV spectrum suggests it is due to the presence of an unidentified phenolic acid (UV *λ*_*max*_ 223 and 332 nm) [38].

### LC-MS analysis

Peak 1 showed a retention time of 7.9 min, UV *λ*_*max*_ of 222 and 326 nm, *m/z* (int.) [M + H]^+^*m/z* 355,1035. The compound 1 was identified as chlorogenic acid after a comparison with the theoretical exact mass of the protonated molecule (calculated as 355.1029, error 1,6 ppm). In addition, the protonated molecule afforded the typical fragment ion [M + H-192]^+^ at *m/z* 163, which is attributed to the loss of quinic acid moiety. The peak 2 showed a retention time of 25.5 min, UV *λ*_*max*_ of 257 and 353 nm, *m/z* (int.) [M + H]^+^*m/z* 611 (protonated molecule) and the fragments at *m/z* 465 and 303. The compound 2 was identified as rutin and its exact mass was calculated as *m/z* 611.1607, (theoretical exact mass: 611.1612 error, 0,8 ppm). The fragments were in agreement with the ion [M + H-146]^+^ at *m/z* 465, which is attributed to the loss of rhamnose moiety and a protonated aglycone ion [M + H-146-162]^+^, whereas the loss of 308 u corresponds to a rhamnose (146 u) plus a glucose (162 u) moiety. The LC-MS analyses were compared with MassBank database (http://www.massbank.jp).

### Evaluation of rutin, chlorogenic acid and aqueous extract of the fruits of Hancornia speciosa in carrageenan-induced peritonitis model

**Fig. 2:**
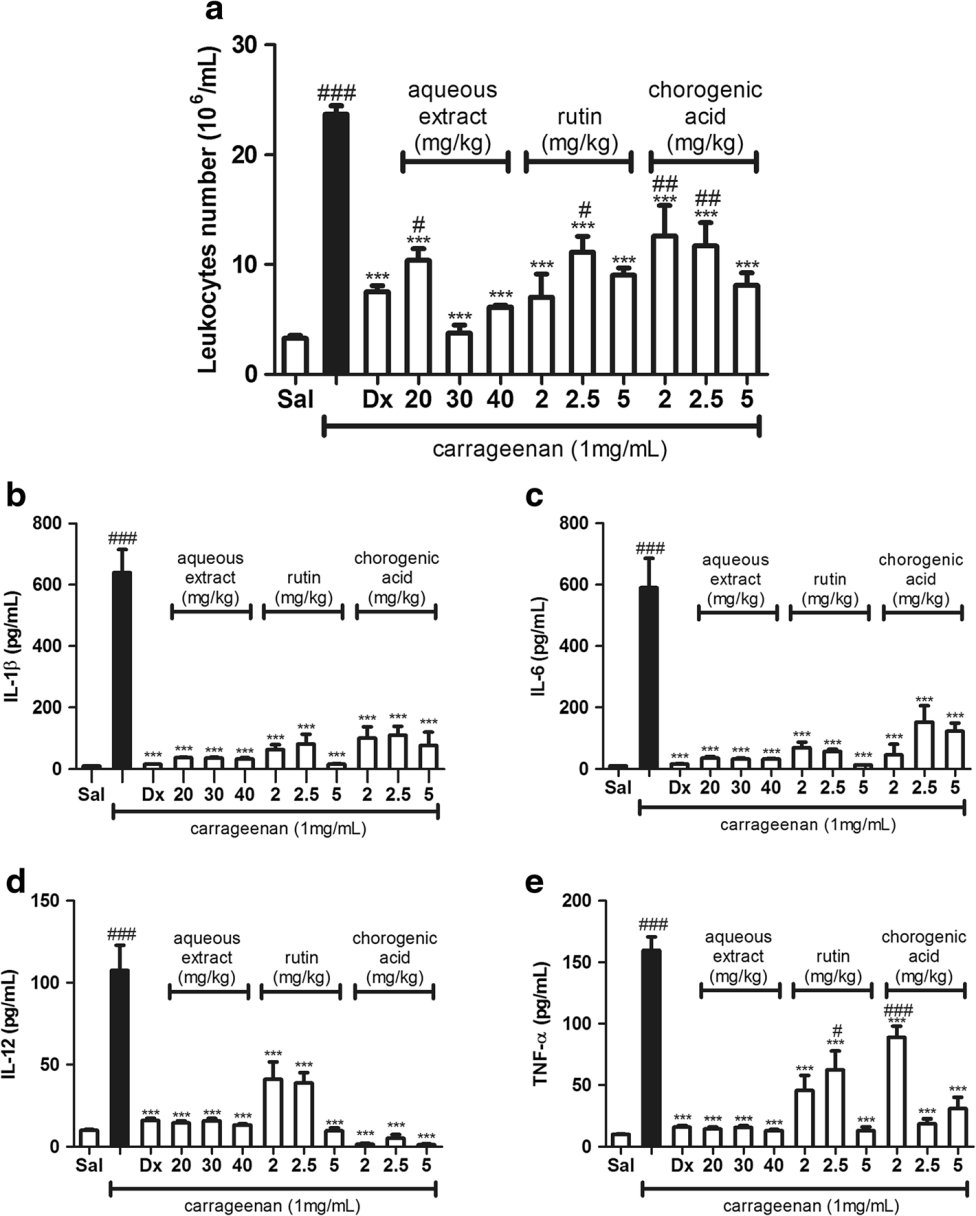
Effect of the aqueous extract from the fruits of *Hancornia speciosa,* rutin and chlorogenic acid in carrageenan-induced peritonitis model. BALB/c mice were treated (i.v.) with saline, aqueous extract at doses of 20, 30 or 40 mg/kg, rutin, chlorogenic acid at the dose of 2, 2.5 or 5 and with dexamethasone (i.p.) at the dose of 0.5 mg/kg, thirty minutes after carrageenan (1 mg/mL) or saline (1 mL) administration (i.p). After 4 h, peritoneal lavage with saline was performed and leucocytes count was performed using a Neubauer chamber (**a**). The supernatants were collected for the determination of IL-1β (**b**), IL-6 (**c**), IL-12 (**d**), and TNF-α (**e**), which was performed using an enzyme-linked immunosorbent assay. The solid column (black bar) represents the groups that received saline solution (i.v.) followed by intraperitoneal administration of carrageenan 30 min later. Each column represents the mean of the values obtained from five animals, and the vertical lines indicate the standard errors of the mean (SEM). ^###^ *p* < 0.001 compared with the group treated with saline; ***p < 0.001 compared with the carrageenan group. Sal: saline (0.9 mg/mL); Dx: dexamethasone (0.5 mg/kg)

The anti-inflammatory activity of the aqueous extract, chlorogenic acid and rutin was evaluated using the carrageenan-induced peritonitis model. As expected, the animals treated intravenously (i.v.) with saline and thirty minutes later with the intraperitoneal (i.p.) administration of carrageenan exhibited a severe cell migration to the peritoneal cavity, as well as an increased production of IL-1β, IL-6, IL-12 and TNF-α. All groups treated (i.v.) with different doses (20, 30 or 40 mg/kg) of the extract, rutin and chlorogenic acid at doses of 2, 2.5 or 5 mg/kg, showed a significant inhibition of cell migration into the peritoneal cavity when compared to those of the groups treated with saline (i.v.) and carrageenan (i.p.) (Fig. 2a). When only the extract (40 mg/kg) was administered intravenously, without the injection of carrageenan, inflammation could not be induced. The different doses (20, 30 or 40 mg/kg) of the extract, as well as rutin and chlorogenic acid at doses of 2, 2.5 or 5 mg/kg, significantly inhibited the production of cytokines IL-1β (Fig. 2b), IL-6 (Fig. 2c), IL-12 (Fig. 2d) and TNF-α (Fig. 2e). In addition, the reduction in the total number of leukocytes and the decrease in the production of cytokines in the group treated with dexamethasone were very similar to those treated with the extract, rutin and chlorogenic acid.

### Evaluation of rutin, chlorogenic acid and the aqueous extract of the fruits of Hancornia speciosa in xylene-induced ear edema model

The effects of rutin, chlorogenic acid and extract on edema formation in mice were investigated using xylene-induced ear edema model. The groups treated (i.p.) with 40, 50 and 60 mg/kg of the extract showed a suppression of the xylene-induced ear edema of 78.36, 81.07 and 73.18 %, respectively. These results were similar to those of the group treated with dexamethasone (i.p.) at a dose of 0.5 mg/kg (79.66 %). When only the extract (60 mg/kg) was administered intraperitoneally, without xylene, it was observed that both ears showed similar weights, with the right and left ears weighing 28.40 mg (±0.8540) and 27.50 mg (±1.067), respectively. These results suggest that the aqueous extract alone is not able to induce the formation of edema. The group treated with rutin (i.p.) received doses of 2.5, 5 and 10 mg/kg, which were able to reduce the edema in 76.94, 83.29 and 97.78 %, respectively, whereas the groups treated (i.p.) with chlorogenic acid with doses of 10, 12.5 and 15 mg/kg showed a reduction of 85.82, 83.17 and 79.84 % of the edema, respectively (Table 1).

**Table 1:** Anti-edematogenic effect of rutin, chlorogenic acid and the aqueous extract from the fruits of *Hancornia speciosa* in xylene-induced ear edema model

Groups (treatment i.p.)	Dose (mg/kg)	Difference (mg)	Inhibition (%)
Saline	----	32.450 ± 2.603	
Dexamethasone	0.5	6.600 ± 2.448***	79.66
Aqueous extract	40	7.020 ± 3.820***	78.36
Aqueous extract	50	6.140 ± 2.385***	81.07
Aqueous extract	60	8.700 ± 2.719***	73.18
Rutin	2.5	7.480 ± 2.221***	76.94
Rutin	5	5.420 ± 2.328***	83.29
Rutin	10	0.720 ± 1.253***	97.78
Chlorogenic acid	10	4.600 ± 2.795***	85.82
Chlorogenic acid	12.5	5.460 ± 2.579***	83.17
Chlorogenic acid	15	6.540 ± 3.917***	79.84

Values are mean ± standard deviation (S.D.), *n* = 5, ***p < 0.001, tested-group compared to saline-treated group

### Evaluation of rutin, chlorogenic acid and the aqueous extract of the fruits of Hancornia speciosa in zymosan-induced air pouch model

**Fig. 3:**
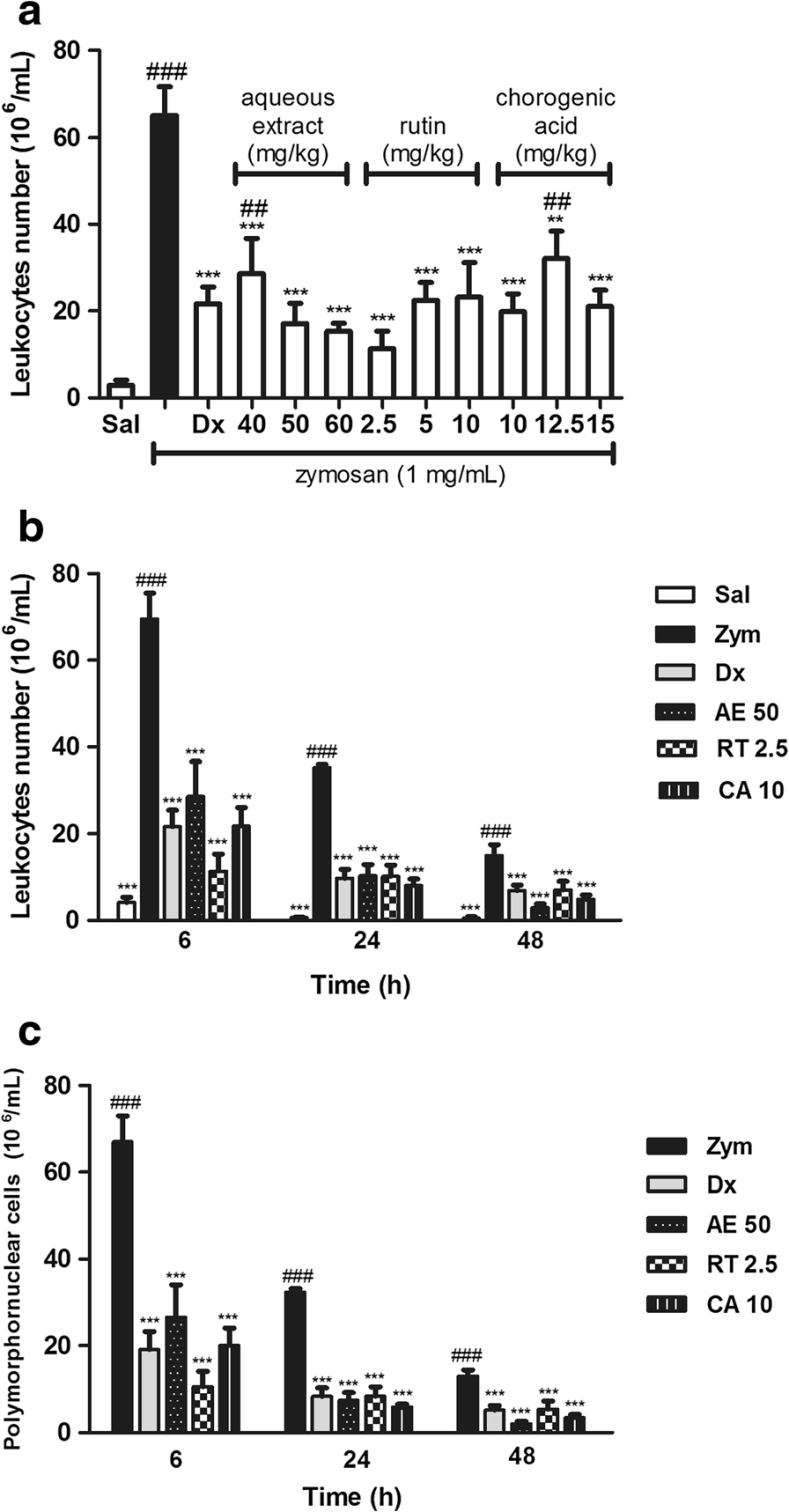
Effect of the aqueous extract from the fruits of *Hancornia speciosa* in zymosan-induced air pouch model. *Swiss* mice were treated (i.p.) with saline, aqueous extract at doses of 40, 50 or 60, rutin, chlorogenic acid at the dose of 2, 2.5 or 5 and with dexamethasone (2.0 mg/kg), thirty minutes after subcutaneous (s.c.) injection of zymosan solution (1 mg/mL) or saline (1 mL) into the animals air pouch. After six hours, the exudate was harvested from each air pouch by lavage with 2 mL of saline and leucocytes count was performed using a Neubauer chamber (**a**). At 6, 24 and 48 h after zymosan administration, the dose of 50 mg/kg of aqueous extract, 2.5 mg/kg of rutin and 10 mg/kg chlorogenic acid were chosen to perform the experiment of leucocytes cell migration kinetics (**b**) and polymorphonuclear cells migration kinetics (**c**). The solid column (black bar) represents the group that received saline solution (i.p.) followed by subcutaneous administration of zymosan 30 min later. Each column represents the mean of the values obtained from five animals, and the vertical lines indicate the standard errors of the mean (SEM). ^###^ *p* < 0.001 compared with the control group (Sal); ***p < 0.001, **p < 0.01 and *p < 0.05, compared with the zymosan group (Zym). Sal: saline (0.9 mg/mL); Zym: zymosan (1 mg/mL); Dx: dexamethasone (2.0 mg/kg); AE: aqueous extract (50 mg/kg); RT: rutin (2.5 mg/kg); CA: chlorogenic acid (10 mg/kg)

After zymosan was injected into the animal’s air pouch, acute inflammation was induced, whose response was characterized by leukocyte migration and plasma exudation to the cavity. Mice treated (i.p.) with saline and thirty minutes later with subcutaneous (s.c.) administration of zymosan showed intense leukocyte migration towards the animals’ pouch. However, all the groups treated (i.p.) with different doses (40, 50 or 60 mg/kg) of the extract showed a significant inhibition of leukocyte migration compared with the group that received only saline (i.p.) and zymosan (s.c.). When only the extract (60 mg/kg) was administered intraperitoneally, without receiving the injection of zymosan, it was observed that cellular migration into the cavity was similar to that of the saline group, which indicates that the extract alone is not able to induce leukocytes migration and hence inflammation. The groups treated (i.p.) with rutin at doses of 2.5, 5 and 10 mg/kg and chlorogenic acid at doses of 10, 12.5 and 15 mg/kg showed a significant inhibition of cell migration (Fig. 3a).

After evaluating the effect of three different doses of rutin, chlorogenic acid and extract, their effects on the kinetics (6, 24 and 48 h) of cell migration and differential count were evaluated using a fixed dose of each of these compounds. The administration of zymosan (s.c.) induced a marked increase in cell migration to the back of the mice at all time points (6, 24 and 48 h). However, the previous treatment with the aqueous extract (i.p.) at a dose of 50 mg/kg, rutin (i.p.) at 2.5 mg/kg and chlorogenic acid (i.p.) at 10 mg/kg was able to significantly inhibit leukocyte migration during all the time points, which was in fact, very similar to that of the group treated with dexamethasone (i.p.) at a dose of 2.0 mg/kg. Thus, the treated-groups showed a marked decrease in the leukocyte migration even 48 h after zymosan inoculation (Fig. 3b). Furthermore, the extract (i.p.) at a dose of 50 mg/kg, rutin (i.p.) at a dose of 2.5 mg/kg and chlorogenic acid (i.p.) at a dose of 10 mg/kg reduced the number of polymorphonuclear cells at all time points (6, 24 and 48 h) (Fig. 3c). The number of mononuclear cells was analyzed, but they did not show statistical difference between groups (data not shown).

## Discussion

In the present study, phytochemical analysis of the extract from the *H. speciosa* fruits indicated the presence of chlorogenic acid and rutin (Fig. 1). The presence of chlorogenic acid has also been demonstrated by [39], where six phenolic acids (chlorogenic, ferulic, gallic, p-coumaric, protocatechuic and vanillic acids) were identified by ultra performance liquid chromatography (UPLC) in the Brazilian tropical fruits “mangaba” (*H. speciosa* Gomes) and “umbu” (*Spondias tuberosa* Arruda Camara) [39]. Rutin and chlorogenic acid have been used for their pharmacological properties such as anti-oxidant, anti-carcinogenic and anti-inflammatory [40–42]. Regarding the anti-inflammatory activity of *Hancornia speciosa*, there is one study that reported this property, but it was performed on its latex [19] and for the best of our knowledge, no study has been performed on its fruits. Our previous in vitro study demonstrated that different concentrations (0.25, 0.375, 0.5, 0.75, 1, 1.25 and 1.75 mg/mL) of aqueous extracts from the fruits of *Hancornia speciosa* did not present significant toxicity in 3 T3 cells. Thus, the data suggest that the doses of *H. speciosa* extracts used in this study are safe. The lack of cytotoxicity effect of *Hancornia speciosa* fruits was corroborated with the cytotoxicity effects of *H. speciosa* latex on the root meristem cells of *Allium cepa* [43].

Thus, the anti-inflammatory effects of the extract of the *H. speciosa* fruits as well as rutin and chlorogenic acid were evaluated using in vivo inflammatory models. The carrageenan-induced peritonitis involves the acute inflammation process that takes place through the release of histamine, serotonin and bradykinin, which lead to an increase in the vascular permeability and prostaglandins production [44, 45]. The increased vascular permeability and higher production of inflammatory mediators resulted in a gradual increase in fluid leakage and a higher number of cells that migrated into the animal’s peritoneal cavity, especially neutrophils, which are capable of producing cytokines that are associated with the inflammatory process. If this process is not controlled, it can cause serious infection that often leads to multiple organ failure, septicemia and mortality [46]. This study showed that the aqueous extract, rutin and chlorogenic acid exerted an anti-inflammatory effect at all tested doses. They were able to reduce cell recruitment into the peritoneal cavity of mice and inhibited the production of cytokines (IL-1β, IL-6, IL-12 and TNF-α). The mechanism involved in their anti-inflammatory activity is still unknown. However, there are hypotheses that can be suggested. Considering that a vascular endothelial cell contraction takes place at the moment of injury, it is likely that an increase in vascular permeability with the production of exudate eventually occurs. Such vascular events induce the activation of inflammatory mediators, followed by the recruitment and adhesion of leukocytes to the inflammation site. These mechanisms are regulated by both cell adhesion molecules and by the production of inflammatory cytokines [21, 47]. Therefore, it seems that the aqueous extract of *H. speciosa* fruits and its bioactive molecules inhibited the inflammatory mediators involved in the carrageenan-induced inflammation, such as histamine, serotonin, kinins and prostaglandins, which are responsible for inducing vasodilatation and formation of exudate in the peritoneal cavity. In addition, they could have inhibited the leukocytes receptors that connect with intercellular adhesion molecules that activate endothelial cells, blocking the migration of the leukocytes to the inflammation site. If these inflammatory mediators and vascular events are blocked or interrupted, the production of inflammatory cytokines will also be compromised.

Induction of ear edema was used as the primary model for acute inflammation, where xylene acted as the phlogistic agent, increasing vascular permeability with edema formation, which is one of the main signs of inflammation. This inflammation process is initiated by the action of mediators such as serotonin, acetylcholine, histamine, bradykinin and prostaglandins, which release neuropeptides that activate its receptors, causing neurogenic inflammation [48]. One of the neuropeptides, called substance P, is a potent vasodilator that acts by releasing nitric oxide from endothelial cells, which causes vasodilatation and plasma exudation, inducing the formation of edema [49]. In this study, it was demonstrated that the extract, rutin and chlorogenic acid were able to reduce at least 73 % of the ear edema, which indicates a promising anti-phlogistic effect. Although the mechanism of action has not been elucidated, it seems that the extract have a membrane-stabilizing effect that reduces vasodilatation, as rutin reportedly improves the strength and integrity of blood vessel walls [50]. The other possibility is through an inhibitory effect over the inflammatory mediators that activate receptors that cause the neurogenic inflammation.

The zymosan-induced air pouch was another in vivo model that was used in this study to investigate the anti-inflammatory properties of the extract, rutin and chlorogenic acid. Due to the fact that the injection of sterile air into the back of an animal forms a cell-lined cavity that resembles the synovial membrane, this model is considered to be similar to the inflammatory response of synovial tissue [36, 51]. Zymosan is a polysaccharide derived from *Saccharomyces cerevisiae* yeasts, whose administration promotes an intense inflammatory reaction [52]. The results revealed that rutin, chlorogenic acid and the extract significantly reduced leukocytes migration to the pouch of the mice at all time points, even after 48 h of zymosan administration. In addition, significant reduction of polymorphonuclear cells, as well as an increase in the number of mononuclear cells shows the ability of the extract to control the inflammation by reducing the number of neutrophils at the inflammation site. The mechanism involved in the inhibition of inflammation by this extract is still unclear. There are reports in the literature that indicate that zymosan interacts with the toll-like receptor 2 (TLR-2). Zymosan is recognized by receptors (dectin-1) present in macrophages, neutrophils and T cells. After the recognition, it interacts with TLR-2. Studies suggest that the combined signaling of dectin-1 and TLR-2 enhance the responses triggered by each receptor [53]. This interaction induces intracellular cascades that activates the selective recruitment of adapter proteins and induces the myeloid differentiation of gene 88 (MyD88), activating the transcription of the nuclear factor kappa B (NF-kB), which is responsible for the transcription of pro-inflammatory genes, resulting in the production of inflammatory cytokines and the expression of co-stimulatory molecules [54]. Previous reports have shown that rutin and chlorogenic acid have several pharmacological activities, especially anti-inflammatory [40, 42]. Recent studies show that both chlorogenic acid and rutin inhibit the activation of NK-kB, suppressing the production of prostaglandin E2 by inhibiting the cyclooxygenase-2 expression [55, 56]. Thus, it seems reasonable to consider that these two secondary metabolites act synergically in this inflammation pathway. Other possibility is that the extract or its bioactive molecules can competitively inhibit the TLR-2 or/and dectin-1 receptor, suppressing the intracellular cascades of inflammation.

Moreover, the inhibition of cell migration towards the inflammation site can not be ruled out as another mechanism for the anti-inflammatory activity of *Hancornia speciosa* fruits. It is known that the inflammatory process occurs through the increase in the vascular permeability, as well through the migration and activation of polymorphonuclear cells, especially neutrophils [57]. Thus, it is possible that the bioactive molecules present in the aqueous extract of *Hancornia speciosa* fruits bind to receptors of endothelial cells, inhibiting the cell migration and the activation of inflammatory mediators involved in chemotaxis and diapedesis.

## Conclusion

In conclusion, these findings demonstrate for the first time that the aqueous extract of the fruits of *H. speciosa* have a marked anti-inflammatory effect in animal models. Moreover, these results suggest that rutin and chlorogenic acid may play a critical role in controlling inflammatory events, contributing, at least in part, for the anti-inflammatory effect of *H. speciosa* fruits. However, additional studies are needed to show that other constituents present in the extract also contribute to the anti-inflammatory effect. Therefore, it seems reasonable to suggest that the extract may represent in the near future a new alternative or complementary option for treating inflammatory disorders.

## Abbreviations

AE, aqueous extract; ANOVA, one-way analysis of variance; CA, chlorogenic acid; DAD, diode array detector; Dx, dexamethasone; ELISA, enzyme-linked immunosorbent assay; ESI, electrospray ionization; HPLC, high performance liquid chromatography; i.p., intraperitoneal; i.v., intravenous; ICAMs, intercellular adhesion molecules; IL-12, interleukin 12; IL-1β, interleukin 1-beta; IL-6, interleukin 6; LC, liquid chromatography; MS, mass spectrometry; MyD88, myeloid differentiation primary response gene 88; NF-kB, nuclear factor kappa B; NSAIDs, non-steroidal anti-inflammatory drugs; PGE2, prostaglandin E2; *Rf,* retention factor; RT, rutin; s.c., subcutaneous; SAIDs, steroidal anti-inflammatory drugs; Sal, saline (0.9 mg/mL); TLR-2, toll-like receptor 2; TNF-α, tumor necrosis factor alpha; Zym, zymosan
